# Structural Brain Correlates of Loneliness among Older Adults

**DOI:** 10.1038/s41598-019-49888-2

**Published:** 2019-09-19

**Authors:** Sandra Düzel, Johanna Drewelies, Denis Gerstorf, Ilja Demuth, Elisabeth Steinhagen-Thiessen, Ulman Lindenberger, Simone Kühn

**Affiliations:** 1 Max Planck Institute for Human Development, Berlin, Germany; 2 Humboldt University Berlin, Berlin, Germany; 3 Charité – Universitätsmedizin Berlin, corporate member of Freie Universität Berlin, Humboldt-Universität zu Berlin, and Berlin Institute of Health, Lipid Clinic at the Interdisciplinary Metabolism Center, Berlin, Germany; 4 Charité - Universitätsmedizin Berlin, Berlin Institute of Health Center for Regenerative Therapies, Berlin, Germany; 5 Max Planck UCL Centre for Computational Psychiatry and Ageing Research, Berlin, Germany; 6 Max Planck UCL Centre for Computational Psychiatry and Ageing Research, London, UK; 7 University Clinic Hamburg-Eppendorf, Hamburg, Germany

**Keywords:** Limbic system, Social neuroscience

## Abstract

Ample evidence indicates that loneliness in old age is associated with poor bodily and mental health. However, little is known about structural cerebral correlates of loneliness in healthy older adults. We examined such correlates in a magnetic resonance imaging (MRI) subsample of 319 older adults aged 61 to 82 years drawn from the Berlin Aging Study II. Using voxel-based morphometry (VBM) and structural equation modeling (SEM), latent hierarchical regression analyses were performed to examine associations of (i) loneliness, (ii) a range of covariates, and (iii) loneliness by covariate interactions with latent brain volume estimates of brain structures known to be involved in processing, expressing, and regulating emotions. Results from whole-brain VBM analyses showed that individuals with higher loneliness scores tended to have smaller gray matter volumes in three clusters comprising (i) the left amygdala/anterior hippocampus, (ii) the left posterior parahippocampus and (iii) the left cerebellum. Significant associations and interactions between loneliness and latent factors for the amygdala and the hippocampus were confirmed with a region-of-interest (ROI)-based approach. These findings suggest that individual differences in loneliness among older adults are correlated with individual differences in the volumes of brain regions that are central to cognitive processing and emotional regulation, also after correcting for confounders such as social network size. We discuss possible mechanisms underlying these associations and their implications.

## Introduction

With increasing life expectancy^1^ older adults are more likely to be confronted with changes in their social network structure^2^. As a consequence, loneliness is on the rise with about 20 to 40 percent of older adults in Western countries reporting to feel lonely today^3^. Loneliness is typically defined as a result of perceiving a discrepancy between the desired quantity and quality of people’s social life and one’s actual social relationships^4,5^. Loneliness can be described as the subjective experience that one’s social network is insufficient in size or unsatisfactory in quality and it has been linked to emotions that need to be regulated to maintain mental and physical well-being^6^. Interestingly, loneliness seems to be a stronger predictor of physical and mental health outcomes than quantitative objective measures of social integration (e.g., social network size, number of friends, marital status, and frequency of contacts^7,8^. In particular, having a large social network does not necessarily prevent the feeling of loneliness^9^.

Lifespan research has long demonstrated that loneliness is associated with key outcomes of aging, including increases in morbidity, overall mortality^3,7,10^, and poorer mental health^9,11–13^. Even though numerous studies have examined behavioral, physical and structural brain correlates of loneliness in relation to specific mental disorders such as anxiety, depression^14,15^ astonishingly little is known about the neural structures and processes associated with feelings of loneliness among healthy older adults^16,17^.

### Loneliness in old age

Feelings of social connectedness are a fundamental human concern^18^ and thus, the subjective experience that one’s social network is sufficient in size or satisfactory in quality is often considered to be a crucial constituent of successful aging^2,4^. Conceptual models suggest that across adulthood and old age numerous social and health factors profoundly shape perceptions of loneliness^19,20^. Because these key sources of loneliness are often accumulating in old age^4^, one could expect that older adults have increasingly fewer opportunities to be socially embedded and integrated, thereby resulting in age-related increases of overall loneliness.

At the same time, it has long been argued that with decreases in remaining lifetime older adults aim to increase their well-being by focusing more on fewer but emotionally meaningful relationships and less on expanding their social networks^21^. For example, when being confronted with an increasing number of losses in ones’ social network, older adults may focus on the most meaningful and important relationships and therefore maintain their overall sense of social embeddedness. As a consequence, loneliness would be expected to remain relatively stable across adulthood and old age.

Empirical studies on age-related changes in loneliness have so far revealed an inconsistent pattern. A number of studies have reported moderate to strong increases in loneliness with increasing age^22,23^; see also the meta-analysis^24^, suggesting that despite older adults’ ability to compensate ageing-related losses, feelings of loneliness emerge as older adults inevitably begin to lose emotionally close relationships^23^. In contrast, other studies did not find associations between loneliness and age, especially when accounting for well-known correlates of loneliness and age (e.g., income^4,25^). These discrepant findings may be caused by study differences in the selection of the samples, its characteristics and size, or measurement and also highlight the need for more fine-grained exploration of the antecedents and consequences of loneliness in old age.

### Loneliness and brain structure

Cacioppo and Patrick (2008) suggested that an increased sensitivity to social disconnection, difficulties in self-regulating one’s emotions associated with feeling isolated, and that the cognitive representations and expectations about others contribute to loneliness. Thus, individual differences in subjective perception of loneliness may in part reflect differences in brain regions that are functionally involved in processing, expressing, and regulating emotionally and socially relevant information^16,26^. Indeed, several findings from functional and structural neuroimaging studies have reported links between specific brain structures and loneliness. It can be assumed that age-related increases in loneliness might be linked to age-related structural changes in brain regions that are in turn related to cognitive processing of emotional and social stimuli and their regulation^16,26^. To shed light onto the current state of research in this field, we reviewed recent studies that aimed to examine neuronal correlates of perceived loneliness. We provide a comprehensive overview of the measures used and reported relevant outcomes of these studies in the Supplementary Information.

For example, in a recent VBM study Kanai and colleagues (2012) showed that individuals reporting higher loneliness showed less gray matter volume of left posterior superior temporal sulcus, a region that has been implicated in processing of social information. Although this study showed that such brain correlates of loneliness may indeed exist, the sample consisted exclusively of younger and middle-aged adults and consequently does not allow to draw conclusions about how these associations evolve in older age. Additionally, the authors did not control for well-known covariates that have been linked to loneliness such as depressive affect, overall physical functioning and personality traits such as openness.

In a different study Sato and colleagues (2016) showed that larger amygdala volume was associated with more perceived social support in a sample of younger adults^27^. However, the authors focused only on associations between perceived social support and amygdala volume, but did not examine the involvement of other covariates of loneliness as well as other brain regions that may be crucial for social and emotional processing.

In contrast, in an intervention study targeting different exercise conditions in older adults D’Agostino, Kattan, and Canli (2018) found a decrease in perceived loneliness which was not related to structural changes in brain regions.

Such discrepant findings could be due to study differences in sample size (ranging from n = 30 to n = 308), sampling strategies (younger vs. older participants), number and type of covariates, or measurement (UCLA loneliness scale vs. NIH Toolbox; see review table in Supplementary Information).

Furthermore, another area of research on brain plasticity has pointed out that changes in environmental demands can shape brain structure, also in the aging brain^28–30^. In recent years, promising evidence from animal models and intervention studies in humans indicates that e.g. the exposure to enriched environments show beneficial effects on structural and neurochemical measures within brain regions that are important for learning and memory such as the hippocampus^28,29,31–33^. One can assume that brain regions that are sensitive to changes in environmental demands and social as well as cognitive stimulation are also more vulnerable to the negative consequences of prolonged loneliness, social disconnection and environmental deprivation^33–36^.

To summarize, conceptual perspectives and empirical evidence have shown that loneliness is associated with specific brain regions and/or networks that are functionally crucial for processing, expressing, and regulating emotionally and socially relevant information^16,26^. These are e.g. brain regions involved in (i) value encoding, e.g. fronto-parietal and anterior cingulate regions and subcortical regions such as the amygdala; (ii) processing emotional, rewarding and motivational features of stimuli such as the ventral striatum, and brain regions that are (iii) vulnerable to environmental deprivation and which may also provide temporal and spatial social contexts related to memory such as the hippocampus. However, empirical studies have typically examined younger age groups and rarely examined the unique role of loneliness over and above well-known confounds and their interaction with loneliness (see also Supplementary Information A.1). Also, the earlier studies noted have investigated associations between loneliness and brain structure using exploratory analyses such as VBM or only focused on one specific brain region such as the amygdala. In our study, we aim to obtain a broader view on the links and interactions between loneliness, the brain and its covariates by combining exploratory and confirmatory analyses instead. Moreover, relatively little is known about whether and how loneliness and its confounders are associated with the aforementioned brain structures in healthy aging.

## The Present Study

Within the present study, we set out to investigate structural brain correlates of loneliness assessed with the UCLA Loneliness Scale within the BASE-II subsample of healthy older participants who additionally underwent MRI.

On the whole brain level we apply VBM in order to exploratory investigate direct association of gray matter volume and loneliness after taking into account important confounders of loneliness. Since this study is based on cross-sectional data a causal directionality of specific effect cannot be inferred. We hypothesized that higher perceived loneliness would be linked to smaller volumes in brain regions that have been implicated in prior studies in emotional and social cognition due to negative consequences of loneliness such as stress, reduced social and cognitive stimulation. In a second set of analyses, we aim to validate in a confirmatory model a latent brain factor model including a priori defined brain regions to jointly observe how loneliness affects these regions. The selection of the ROIs was based on findings from the current literature on brain structure and loneliness (see Supplementary Information). Our goal was to explore possible moderation and interaction effects of a number of potential confounders that have been previously shown to be associated with loneliness and brain structure, namely age, gender, years of education, depressive affect, openness, morbidity, total intercranial volume (TIV) and number of confidants^3,15,37^.

To our knowledge our study is the first to investigate in an exploratory and confirmatory fashion these associations in healthy older adults.

## Results

Table 1 reports intercorrelations for the variables under study. Higher loneliness is associated with younger age, being a woman, and fewer years of education as well as fewer confidants, less openness, more depressive effect, and higher morbidity (all p’s < 0.05). Table 2 summarizes the intercorrelations between the latent brain factors and covariates resulting from the SEM analyses. Most latent brain factors show a negative correlation with age (all p’s < 0.05), gender, education and TIV. There were no significant associations found between depressive affect, openness, or morbidity and the latent brain factors.

**Table 1:** Descriptive statistics and intercorrelations for all variables under study of the full sample.

	Intercorrelations									
	mean	SD	Age	Gender	Education	Loneliness	Number of Confidants	Depressive Affect	Openness	Morbidity
Age (61–88 years)	70.1	3.7	1							
Gender (1 = men, 2 = women)	1.51	0.49	0.19	1						
Education (8.5–18)	14.20	2.87	−0.06*	−0.13*	1					
Loneliness (1–5)	1.56	0.62	−0.08**	−0.05*	−0.06*	1				
Number of confidants (0–18)	4.75	4.40	−0.05	−0.05	−0.01	−0.26**	1			
Depressive Affect (1–5)	2.26	0.72	−0.02	0.04	−0.04	0.36**	−0.13**	1		
Openness (1–7)	5.03	1.17	−0.07**	−0.05	−0.04	−0.18**	−0.12**	−0.10**	1	
Morbidity (0–5)	1.24	1.31	0.01	−0.05*	−0.03	−0.06*	0.05	0.15**	−0.03	1

*p < 0.05; **p < 0.01.

**Table 2:** Intercorrelations of the latent brain factors and covariates resulting from the SEM analyses.

Latent Brain Factor	Covariates						
	Age	Gender	Education	Openness	Depressive Affect	Morbidity	TIV
moFC	−0.15*	−0.11	0.16**	−0.01	−0.01	−0.03	0.38**
dlPFC	−0.16*	−0.01	0.18**	0.11	−0.09	−0.003	0.35**
Amygdala	−0.19*	−0.23*	0.15*	−0.03	−0.08	−0.02	0.01
Hippocampus	−0.38**	−0.21*	0.11	−0.12	−0.10	−0.07	−0.02
Insula	−0.13	−0.28**	0.15*	0.04	−0.05	0.03	0.30**
Nacc	−0.20**	−0.28**	0.21**	0.04	−0.12	0.03	0.17
ACC	−0.18*	−0.06	0.23**	0.01	0.04	−0.01	0.31**

mOFC = medio-orbito prefrontal cortex, dlPFC = dorso-lateral prefrontal cortex, Nacc = Nucleus Accumbens,

ACC = anterior cingulate cortex, TIV = total intercranial volume. *p < 0.05; **p < 0.01.

**Figure 1:**
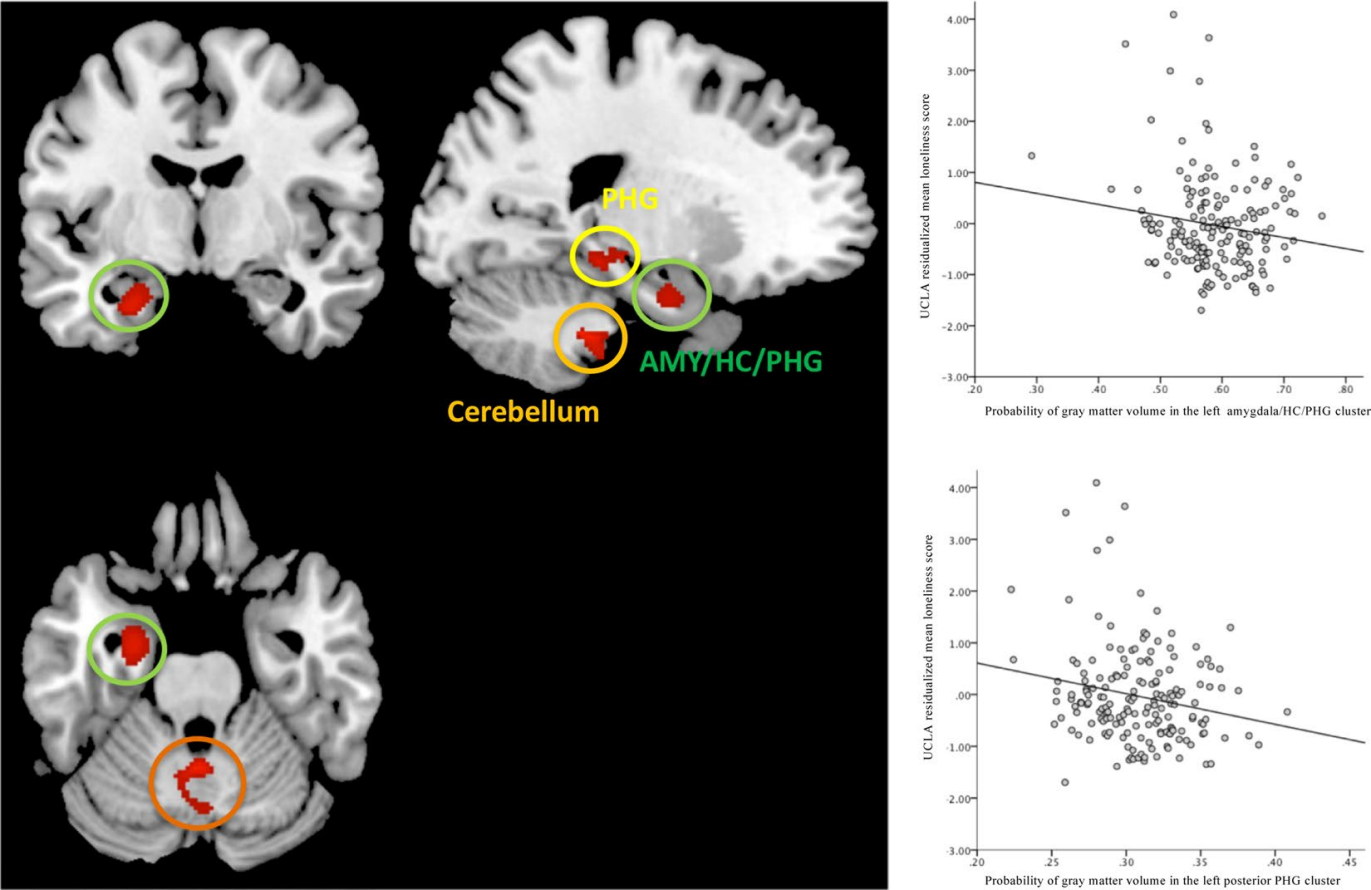
Left: Brain regions showing a negative association (red clusters) between loneliness and gray matter volume (p < 0.001; corrected for multiple comparisons; cluster extent threshold k > 97). Upper right panel: Correlation between the individual GM volumes in the first cluster and loneliness score. Lower right panel: Correlation between the individual GM volumes in the second cluster and loneliness score (p < 0.05; all scores are residualized for covariates).

The VBM analysis revealed three significant clusters (Fig. 1). The first cluster extended from the left anterior amygdala, hippocampus, into parts of the left parahippocampal cortex (peak voxel: x = –27, y = –7, z = –25; p < 0.001; Fig. 1 left; p < 0.001), left posterior parahippocampal gyrus (peak voxel: x = –21, y = –33, z = –12), and the left cerebellum (peak voxel: x = –25, y = –35, z = –44; p < 0.001) after correcting for multiple comparisons (cluster extent threshold k > 97) and controlling for the selected covariates. The right panel of Fig. 1 depicts the three significant clusters resulting from VBM analyses, suggesting that smaller volume in these brain structures is correlated with a higher degree of perceived loneliness. A post-hoc VBM analyses we additionally investigated possible associations between social network size and brain structure that revealed no significant cluster when taking into account the same covariates.

### Factorial structure of the latent brain factor model

**Figure 2:**
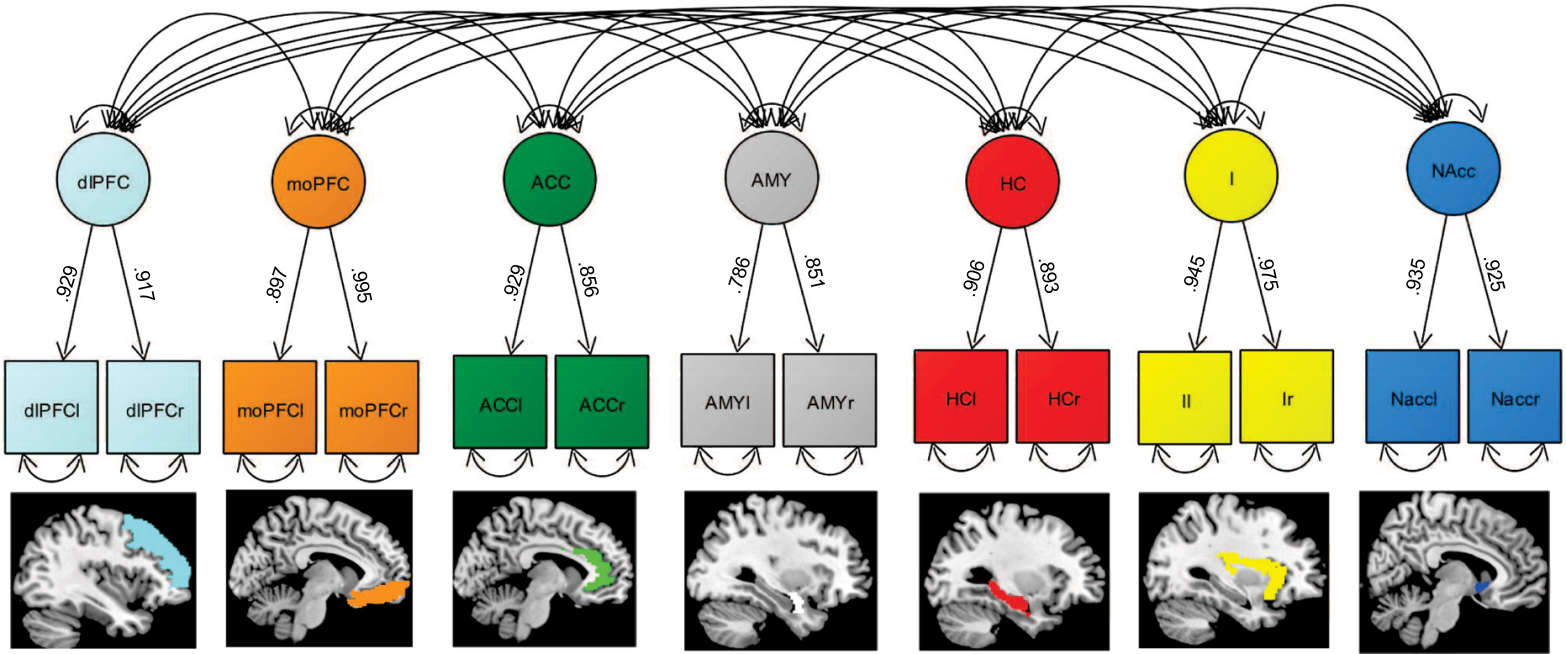
A simplified illustration of the CFA of the latent brain factor model. Model fit is good (χ2 56 = 174.3, RMSEA = 0.058 (CI: 0.044–0.073); CFI = 0.979; SRMR = 0.02). Latent brain factors are drawn in circles. Squares represent observed variables. Single-headed arrows represent significant factor loadings, and double-headed arrows represent covariances. Double-headed arrows with both heads pointing on a manifest variable represent the variance of a variable.

Core results of the CFA for the intercorrelated seven latent brain factor model are shown in Fig. 2. The data fitted the postulated model well (model fit: χ^2^ 54 = 81.3, RMSEA = 0.045; CFI = 0.991; SRMR = 0.02, Fig. 2). All items loaded reliably on the postulated latent factors (standardized loadings > 0.500, p < 0.001). Moreover, most latent factors revealed a moderate to high covariance (Table 3). As a consequence, we used this latent brain factor model as baseline model to investigate brain-loneliness associations in more depth.

**Table 3:** Standardized intercorrelations between each of the latent brain factors resulting from the CFA analyses and results of the SEM reporting the standardized coefficients of loneliness and loneliness by age interactions on each latent brain region.

Latent Brain Factors	moFC	dlPFC	Amygdala	HC	Insula	Nacc	ACC	loneliness	Interaction lone*age
moFC	1							−0.10*	0.19*
dlPFC	0.84**	1						−0.43*	0.34*
Amygdala	0.53**	0.56**	1					−0.15*	0.27*
Hippocampus	0.50 **	0.55**	0.92**	1				−0.49*	0.46*
Insula	0.72**	0.83**	0.58**	0.56**	1			−0.31*	0.20*
Nacc	0.58**	0.63**	0.54**	0.52**	0.61**	1		−0.18*	0.12*
ACC	0.80**	0.83**	0.56**	0.50**	0.69**	0.67**	1	−0.44*	0.49*

mOFC = medio-orbito prefrontal cortex, dlPFC = dorso-lateral prefrontal cortex, HC = hippocampus, Nacc = nucleus accumbens, ACC = anterior cingulate cortex.

*p < 0.05, **p < 0.01.

**Figure 3:**
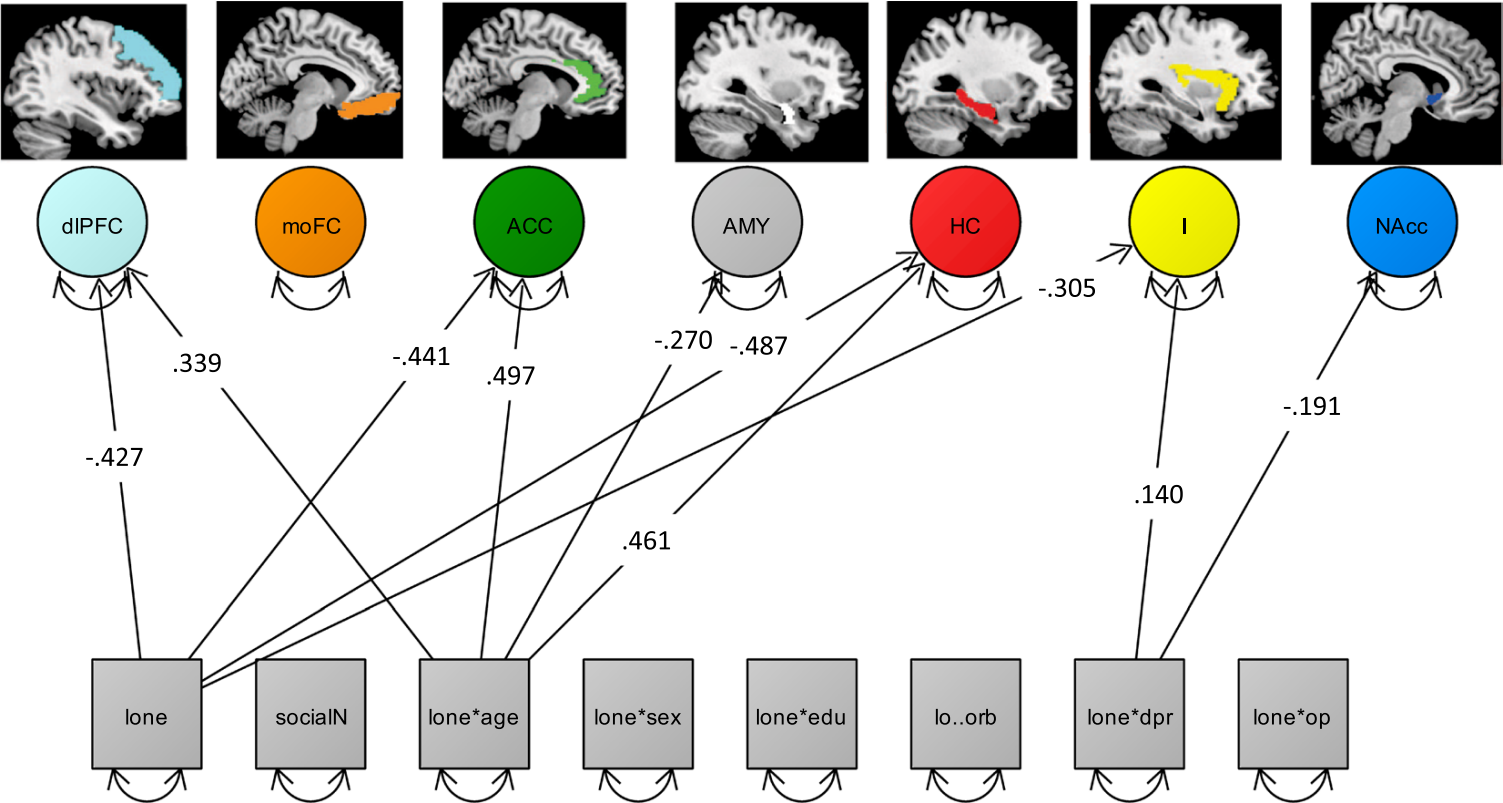
Depiction of a simplified SEM (χ2 159 = 249.8, RMSEA = 0.047; CFI = 0.965) showing only all significant main effects of loneliness (lone) and number of confidants (social) as well as the interaction of age (lone*age), sex (lone*sex), education (lone*sex), morbidity (lo*orb), depression (lone*dpr) and optimism (lone*op) with loneliness, which are regressed on the intercorrelated latent brain factors (circles, intercorrelations not shown). Squares represent observed variables. Double-headed arrows with both heads pointing on a manifest variable represent the variance of a variable. Loneliness, depressive affect, openness and educations are represented as mean scores (squares). dlPFC = dorso-lateral-prefrontal cortex, mOFC = medio-orbito-prefrontal cortex, ACC = anterior cingulate cortex, AMY = amygdala, HC = hippocampus, I = insula, and NAcc = nucleus accumbens. Regression coefficients represent standardized estimates.

Next, we set up a latent regression model to examine simultaneous associations of the individual loneliness scores and the latent brain factor model, whilst simultaneously investigating the main effects as well as the interactions with loneliness and its covariates. The model fitted the data well (χ_2_ 152 = 179.1, RMSEA = 0.030; CFI = 0.990; Fig. 3).

Moreover, observing the regression paths, we found negative main effects of age on all brain regions, except HC and I (p’s < 0.05). Negative main effects of loneliness on dlPFC (β = −0.43, p = 0.02), HC (β = −0.49, p = 0.01), I (β = 0.35, p = 0.04), ACC (β = 0.44, p = 0.01). Regarding the associations between loneliness and covariates (not depicted in Fig. 3) a positive main effect of education on dlPFC (β = 0.18, p = 0.005), AMY (β = 0.15, p = 0.04), I (β = 0.15, p = 0.02), Nacc (β = 0.21, p = 0.001), and ACC (β = 0.23, p = 0.00) were found. Sex showed a negative main effect on AMY (β = −0.23, p = 0.03), HC (β = −0.21, p = 0.04), I (β = −0.28, p = 0.002), and Nacc (β = −0.28, p = 0.005). TIV showed a positive main effect except on all brain regions (all p’s < 0.05), except AMY, HC, and Nacc. Significant loneliness by age interactions were found for dlPFC (β = 0.339, p = 0.04), AMY (β = 0.17, p = 0.04), HC (β = −0.38, p = 0.02), and ACC (β = 0.49, p = 0.005). Loneliness by depression interaction were revealed in dlPFC (β = 0.18, p = 0.02), I (β = 0.14, p = 0.04).

**Figure 4:**
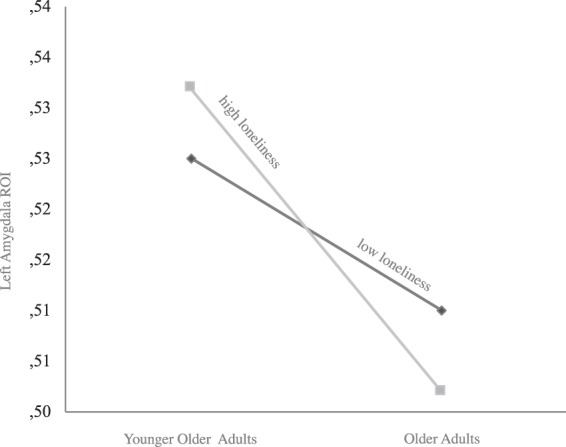
Exemplary plot of the interaction between loneliness, age, and left amygdala volume. ROI are extracted from CAT12 preprocessed gray matter maps. For illustration, the participants were divided into two age groups by applying a 69.8 split for age and loneliness across the sample (median age = 69.8 years; younger older adults  < 69.8 years, older adults = >69.8 years; low loneliness = −1 SD, high loneliness = +1 SD).

Figure 4 exemplarily illustrates the interaction observed between loneliness and age for the left amygdala ROI extracted via CAT12. It can be obtained that older adults reporting a higher level of perceived loneliness show significantly lower volumes in specific brain regions such as the amygdala (depicted in Fig. 4) and in dlPFC, HC, and ACC (not depicted here).

## Discussion

In the present study we applied multivariate methods to identify associations between individual differences in loneliness and regional brain volumes in a sample of healthy older adults. An additional aim of this study was to explore interactive effects of potential confounders of loneliness on these associations and to disentangle the effects of perceived loneliness and social network size regarding their specific associations with brain structure. An exploratory whole-brain VBM analysis revealed that individual differences in loneliness significantly predicted GM volume in one cluster, comprising left amygdala, anterior hippocampus, and adjacent entorhinal/parahippocampal cortex. Another significant cluster evolved in the left posterior parahippocampal gyrus, and in the cerebellum, after taking a comprehensive set of confounders into account (Fig. 1). The VBM results show that higher levels of loneliness are associated with smaller volumes in these regions.

These findings are consistent with previous studies in animals and human young adults showing that the amygdala plays an important role in emotion processing and social functioning (e.g.^38^). Interestingly, previous studies have shown that the GM volume of the amygdala among other structures is correlated with real-world social network size as well as online social network size^26^. In contrast to these studies that focussed on objective measures of social connectedness, we applied the UCLA Loneliness Scale as a commonly used and validated measure of perceived loneliness and controlled for number of confidants.

However, the directionality and etiology of these associations found in the current study  remain unclear. For instance, individuals who have encountered more social stimulation and interactions throughout their life may have enlarged their amygdala volumes, or individuals with larger amygdala to begin with may be more socially inclined.

Moreover, our VBM results point towards a relation between loneliness, hippocampal and parahippocampal regions in healthy aging. Two possible explanations which are not mutually exclusive need to be resolved in future studies. First, higher levels of stress induced by prolonged loneliness could lead to hippocampal injury due to elevated levels of glucocorticoids, and blood pressure^39–42^. Second, loneliness is associated with deprivation of social interactions and then the associated impoverishment of hippocampal drive could lead to atrophy in these regions. One may assume that individuals reporting higher loneliness show a reduced engagement in an active, socially and cognitively stimulating lifestyle which has been repeatedly shown to be crucial to successful cognitive and brain aging^43–45^.

On the other side, it has been shown in lesion studies that damage in the amygdala impairs the ability to recognize emotional facial expressions^46,47^. Consistent with this lesion finding, a previous functional MRI (fMRI) study supports the association between amygdala activation and emotion processing because amygdala activity in response to negative emotional facial expressions and increased anxiety was associated with perceiving more social support^48^. Another fMRI study used a modified emotional Stroop task, in which lonely participants showed greater Stroop interference effects specifically for negative social as compared to negative nonsocial words and to the non-lonely participants^49^. These fMRI findings suggest that loneliness may increase attention to negative social stimuli and vice versa (e.g., social threats). Thus, the hypersensitivity to negative social information and the diminished pleasure derived from positive social stimuli found in the aforementioned studies might shape social expectations and motivations and contribute to higher perception of loneliness. This disposition, in turn, may increase age-related structural changes in the underlying neurobiological systems such as in the amygdala.

Interestingly, VBM analysis additionally revealed a significant cluster in regions of the left cerebellum. The cerebellum has previously been suggested to play a role not only in motor behavior but also in multiple domains of cognitive functioning and emotional control (see^50,51^). The cerebellar connections to the brainstem, limbic system, and PFC may indicate the cerebellum’s significance for emotion processing and its potential role as a central hub for the different information streams.

In a next set of analyses, we applied a latent factor model of a priori selected brain regions in order to test their simultaneous association with loneliness and selected covariates using CFA. By establishing latent factors of brain regions the dimensionality of the data can be reduced and the shared variance can be captured while measurement error is taken care of. As a result we achieved a good model fit for the data thus, validating the latent brain factor model.

 As a consequence, we applied SEM to test the effects of loneliness and its covariates such as age, depressive affect, number of confidants, onto structural integrity in the a priori defined latent brain regions. The SEM results revealed negative main effects of loneliness specifically on dlPFC, HC, I, and ACC.

In line with our findings these regions have been shown to be involved in neural processes that are recruited when experiencing physical pain but also when experiencing social deprivation^52^. Whereas the ACC is more implicated in self reflectional processes^53^ which acts as a neural conflict monitor, detecting conflicts with current goals. In addition, large parts of the dlPFC comprise a brain region that is commonly described as ventromedial prefrontal cortex. This is a key region of central midline structures that have been consistently related to brain activation while participants process stimuli that are strongly related to themselves^54,55^.

The frontoparietal executive networks have been implicated in conscious emotion regulation and reappraisal, whereas the ACC, I, and Amy are rather implicated in emotional reactivity. In general, studies have found that reappraisal of negative emotion activates dorsal ACC and PFC systems that support the selection and application of reappraisal strategies, and decreases, increases or maintains activity in appraisal systems such as the amygdala or insula in accordance with the goal of reappraisal^56^.

Furthermore, SEM revealed specific loneliness by age interactions with dlPFC, Amy, HC, and ACC volumes (Fig. 3) showing that older adults reporting a higher level of perceived loneliness linked to lower volumes in these brain regions. In contrast, younger older adults reporting higher loneliness showed higher volumes within these brain regions. This interaction suggest that the negative effect of loneliness is stronger with advanced age.

## Limitations

The present study has a set of limitations. First, associations between loneliness and brain structure are cross-sectional in nature, therefore the temporal direction let alone causality remains unclear. Longitudinal designs and intervention studies are needed to provide more conclusive evidence on lead-lag relations. Furthermore, the role of possible moderators that might influence the strength of associations requires further investigations in longitudinal studies. For example, previous studies have highlighted the importance of depression in associations between loneliness and brain structure^17^ suggesting that increases in depressive episodes over time could moderate associations between changes in brain structure and perceptions of loneliness. It is also difficult to assess individuals’ social networks objectively. Our assessment by means of one single item asking for the number of confidants leaves room for interpretation. Responses may well be influenced by differences in expectations and social desirability or cognitive functioning of the respondent.

## Conclusion

To our knowledge, this is the first evidence linking GM volume of the left anterior amygdala, hippocampus, parahippocampal gyrus and cerebellum to perceived loneliness after controlling for a substantial set of covariates as well as number of confidants. We used a multivariate approach to analyze associations between self-reported loneliness and objective measures of brain structure in a cross-sectional study. We take our results to illustrate that subjective perception of social embeddedness not only plays a crucial role for health, psychosocial functioning, and well-being reported in several former studies but is also associated with individual differences in volume of specific brain regions. Our results underline the importance of perceived loneliness as a construct independently of social network size in healthy aging that is linked to individual differences in specific brain structures that are involved in emotion processing and memory, even after controlling for other covariates. The results highlight that efforts to promote or maintain brain plasticity in aging are closely intertwined with subjective factors such as loneliness or motivation, conceived as putative antecedents, correlates, or consequences of age-related brain changes.

Longitudinal studies and intervention studies are needed to elucidate the causal network underlying these multivariate associations of loneliness and neural plasticity.

## Methods

### Participants and study design

Participants were recruited from BASE-II, a multi-institutional study capturing multidisciplinary variables from a wide range of domains for each participant^57,58^. Older participants in the BASE-II study ranged in age from 61 to 88 years (n = 1,591; mean = 70.1; SD = 3.7; 50.9% female). None of the participants took any medication that may have affected memory function or had a history of head injuries, medical (e.g., myocardial infarction), neurological, or psychiatric disorders (e.g., epilepsy or depression). All participants had completed at least eight years of education and reported normal or corrected to normal vision, were right-handed, and scored more than 27 points on the MMSE^59^. Individuals participated in two cognitive testing sessions scheduled one week apart. In between these sessions measures of loneliness, openness, and depressive affect were obtained as part of a take-home questionnaire.

Next, eligible participants were invited to a structural brain imaging session within a mean time interval of 3.2 months after completing the cognitive testing and psychosocial assessment. The MRI subsample consisted of 319 older adults aged 61–81 years (mean age = 70.0 years; SD = 3.7 years; 38.3% female). The MRI subsample did not differ greatly from the full BASE-II sample. The largest significant difference was found in sex distribution, with 38% females in the MR-sample and 51% females in the full sample. The Ethics Committee of the Max Planck Institute for Human Development approved the procedure, and the Ethics Committee of the German Society for Psychology (DGPs) additionally approved of the MRI protocol. Participants provided written informed consent and received monetary compensation for their participation in BASE-II and the MRI study. Participants provided informed consent in accordance to the Declaration of Helsinki.

### Measures

*Loneliness*. Loneliness was measured by means of the UCLA Loneliness Scale^60^ calculated as a mean score of seven items. The scale contained such items as “There are people I feel close to” or “I feel isolated from others”. Participants were asked to rate each statement on a 5-point Likert scale ranging from 1–5 (‘1 – strongly disagree’ to ‘5 – strongly agree’). Higher scores indicate stronger feelings of loneliness (Cronbach’s α = 0.81^61^).

### Covariates

#### Socio-demographic variables

Participants’ *age* was calculated as years from birth. *Gender* was indicated by a dichotomous variable (1 = male; 2 = female). *Education* was indicated by the number of years in formal schooling (range: 8.5–18).

#### Social network size

Social network size was obtained with one item asking for the total number of confidants, ranging from 0–18 in our sample.

#### Depressive affect

To assess depressive affect, we used a 3-item subscale of the Positive and Negative Affect Schedule (PANAS-X^62^). Internal consistency was acceptable, with Cronbach’s α = 0.64.

#### Openness

To assess the personality disposition of openness, we used a 3-item subscale of the short version of the Big Five Inventory (BFI^63,64^). Participants were asked to indicate their agreement on a 7-point Likert scale (‘1 = does not apply at all’ to ‘7 = applies perfectly’). Internal consistency was modest (Cronbach’s α = 0.52), but comparable to other reports using few heterogeneous items to measure relatively broad constructs^65,66^.

#### Morbidity

As part of the BASE-II medical examination at the Charité Universitätsmedizin Berlin, diagnoses were obtained through participants’ reports, with selected diagnoses (e.g., diabetes mellitus) being verified by additional laboratory tests (for details, see^57^). These medical diagnoses were used to compute a morbidity index largely based on the categories of the Charlson Comorbidity Index^67^. This is an age-weighted sum of moderate to severe, mostly chronic physical illnesses, including cardiovascular (e.g., congestive heart failure), cancer (e.g., lymphoma), and metabolic diseases (e.g., diabetes mellitus; for details, see^68^).

#### Total Intracranial Volume (TIV)

A well-known source of individual differences in total and regional brain volume is the between-person variation in head size^69^, often measured by TIV. For example, some of the differences between the sexes in brain volume can be accounted for by differences in TIV^70^. We used the probabilistic tissue class images derived from CAT12 segmentation to give tissue volume estimates, with TIV simply being the sum of gray matter, white matter, and CSF voxels.

#### Time interval between MRI and medical session

Data used to index morbidity were collected prior to the MRI sessions (mean interval in years = 1.2 years; SD = 0.80). To control for individual differences in delay between the two measurements, we included the interval as a control variable in the analysis (difference date A minus date B).

### MRI acquisition

#### Scanning procedure

The structural images were acquired on a Siemens Tim Trio 3 T scanner (Erlangen, Germany) using a 32-channel head coil. The T1 images were obtained using a three-dimensional T1-weighted magnetization prepared gradient-echo sequence (MPRAGE) based on the ADNI protocol (www.adni-info.org, repetition time (TR) = 2500 ms; echo time (TE) = 4.77 ms; TI = 1100 ms, acquisition matrix = 256 × 256 × 176, flip angle = 7°; 1 × 1 × 1 mm voxel size).

#### VBM preprocessing

Structural data were processed with CAT12 (Computational Anatomy Toolbox 12) of the Structural Brain Mapping group, Jena University Hospital, Jena, Germany, a toolbox which is implemented in SPM12 (Statistical Parametric Mapping, Institute of Neurology, London, UK) for voxel-based morphometry analysis of imaging data. We applied the CAT12 default cross-sectional preprocessing stream which implements correction of the T1-weighted images for bias-field inhomogeneities, segmentation into grey matter (GM), white matter (WM) and cerebrospinal fluid (CSF), and spatial normalized using DARTEL algorithm. GM images were used for the current set of analyses and thus, smoothed with a Gaussian kernel of 8 mm (FWHM). In order to exclude artefacts on the grey-/white-matter border caused e.g. by incorrect voxel classification, we applied an absolute grey-matter threshold of 0.1.

#### ROIs Extraction

Based on prior functional and structural MRI studies exploring brain regions that are involved in emotion processing and loneliness we extracted ROIs from the medio-orbitofrontal cortex (mOFC), dorso-lateral prefrontal cortex (dlPFC), anterior cingulate cortex (ACC), insula (I), amygdala (Amy), hippocampus (HC), as well as the nucleus accumbens (NAcc) as defined by the automated anatomical labeling atlas^71^. All selected ROIs were extracted from the left and right hemisphere from individual GM maps created by means of CAT12 preprocessing stream.

### Data Analyses

Sample descriptives were computed using SPSS 22.0 (SPSS Inc., Chicago, IL, USA).

#### VBM analyses

Statistical analysis was carried out by means of VBM in SPM12 as a whole-brain multiple regression of GM volume and the individuals’ loneliness score. Age, sex, education, depressive affect, openness, morbidity, time interval between the two sessions, number of confidants, and TIV were entered as covariates. The resulting maps were thresholded with p_uncorrected_ < 0.001. We applied correction for multiple comparisons by using ‘SPM’s nonstationarity correction’ from CAT12 combined with a non-stationary smoothness correction based on permutation as proposed by Hayasaka and Nichols^72^.

#### Structural equation modeling (SEM)

SEM is a multivariate statistical approach, which attracts increasingly attention in psychology and neurosciences^73–75^. It allows testing structural hypotheses about associations and influences among multiple variables by examining how well a given model is able to reproduce the variance–covariance matrix of a set of observed variables. A SEM model typically consists of a measurement model, specifying the relationship of a number of observed variables to latent (unobserved) variables (cf.^76^). The advantage of using latent variables is that they can be assumed to be free of task-specific sources of variance as well as measurement error which is a robust measure of underlying processes or tissue characteristics in contrast to focus on single observed measures. With these considerations, we applied a CFA to establish how well a latent factor model defined by intercorrelated volumes of specific brain regions can be reproduced by the variance–covariance matrix of the observed data. In this study, the CFA and SEM were established by using MPlus v6.1^77^. To ease optimization, all observed scores were z-standardized correcting for inter-variable differences in scaling. Structural models are evaluated by their model fit. Thus, we relied on standard indices such as the Root Mean Square Error of Approximation (RMSEA) and the Comparative Fit Index (CFI). Commonly accepted thresholds indicating good model fit are 0 < = RMSEA < = 0.05 and 0.97 < = CFI < = 1^78^. Models were fitted with full information maximum likelihood estimation (FIML). Following usual practice by implementing FIML in SEM individuals with missing data can be included in the model without imputing missing data.

In a first step we set up a CFA comprising of seven intercorrelated latent factors consisting of pre-selected brain regions, mOFC, dlPFC, ACC, I, Amy, HC, and NAcc. For each corresponding region the latent factor was defined by two indicators, namely the mean of the signal extracted from left and right gray matter maps originating from CAT12 cross-sectional preprocessing stream. All latent factors were defined as correlated dependent variables.

Next, a multiple regression analysis was performed in SEM to estimate how loneliness, its confounders, as well as its interactions were associated with the latent brain factor model. We predicted that higher loneliness is associated with smaller volumes in the pre-defined latent brain regions. In this model, the mean UCLA loneliness score was simultaneously regressed on all intercorrelated latent brain factors. The reported correlations and path coefficients refer to the standardized solution also including the correlations and interactions with age, sex, education, depressive affect, openness, morbidity, whole brain volume, time interval between sessions and number of confidants serving as potential moderators of these associations.

## Supplementary information

Supplementary Information

## Data Availability

Data can be requested from the steering committee of the Berlin Aging Study II. Further Information can be found under https://www.base2.mpg.de/en
