# Supplementary material for: Structural Brain Correlates of Loneliness among Older Adults
Source: Sci Rep. 2019 Sep 19;9:13569. doi: 10.1038/s41598-019-49888-2 (PMC6753249; doi:10.1038/s41598-019-49888-2)
Supplement: Supplementary file 1 — Supplementary Information [file 41598_2019_49888_MOESM1_ESM.pdf]

Structural Brain Correlates of Loneliness among Older Adults

Sandra Düzel[1]*, Johanna Drewelies[2], Denis Gerstorf[2], Ilja Demuth[3], Elisabeth Steinhagen-Thiessen[3], Ulman Lindenberger[1, 5] & Simone Kühn[1,6]

[1]Max Planck Institute for Human Development, Berlin, Germany

[2]Humboldt University Berlin, Germany

[3]Charité – Universitätsmedizin Berlin, corporate member of Freie Universität Berlin, Humboldt-Universität zu Berlin, and Berlin Institute of Health, Lipid Clinic at the Interdisciplinary Metabolism Center, Germany

[4]Charité - Universitätsmedizin Berlin, BCRT - Berlin Institute of Health Center for Regenerative Therapies, Berlin, Germany

[5]Max Planck UCL Centre for Computational Psychiatry and Ageing Research, Berlin, Germany, and London, UK

[6]University Clinic Hamburg-Eppendorf, Hamburg, Germany

Supplementary Information

A.1 Review of Studies investigating Associations between Loneliness and Size and Activation of Different Brain Regions

author, year of publication	journal	waves	n	age-range	measure of loneliness	covariates
Cross-sectional Studies						
Layden et al., 2016	*NeuroImage*	1	55	20-29	Revised UCLA Loneliness Scale (Russell, 1996)	age; gender; depressive symptomatology; objective isolation
Inagaki et al., 2017	*Social Cognitive and Affective Neuroscience*	1	31	24.26 ± 7.57	Revised UCLA Loneliness Scale (Russell, 1996)	age; gender; ethnicity; feelings of social connection
Kanai et al., 2012	*Current Biology*	1	108	18-39	UCLA Loneliness Scale (Russell et al., 1980)	age; gender; social network size; anxiety; empathy; social perception skills; autistic traits
Kong et al., 2014	*Experimental Brain Research*	1	308	18-27	Revised UCLA Loneliness Scale (Russell, 1996)	age; gender; total GMV; neuroticism; extraversion; psychoticism

Tian et al., 2017	*PLoS ONE*	1	30	21.3 ± 2.4	UCLA Loneliness Scale (Russell et al., 1978)	anxiety; depression; interpersonal reactivity; trust; social support
Ti et al., 2018	*Social Cognitive and Affective Neuroscience*	1	100	22-35	NIH Toolbox (Salsmann et al., 2013)	perceived emotional support; perceived instrumental support
Wong et al., 2016	*Psychological Medicine*	1	54	67.45 ± 5.427	20-item UCLA-Loneliness Scale (Russell 1996)	age; gender; MMSE; Depressive symptoms; Severity of depression, onset age (years); number of episodes; actual depressed (months); use of hypnotics (months)
D'Agostino, Kattan, Canli, 2018	*Social Neuroscience*	1	99	18-22; 57-69:	UCLA Loneliness Scale (Russell et al., 1978)	age; social network (The Social Network Index (SNI))
Sin et al., 2017	*International Journal of Geriatric Psychiatry*	1	52	67.92 ± 5.0	20-item UCLA-Loneliness Scale (Russell 1996)	age; gender; Hamilton Rating Scale for Depression (HAMD); Short-Form Health Survey (SF-36)
					Longitudinal Studies	
Ehlers et al., 2017	*Frontiers in Aging Neuroscience*	2	247	60-79	UCLA Loneliness Scale (Russell et al., 1980)	perceived social support; perceived stress; gender; race; education; marital status; weight

Study	Findings – greater self-reported loneliness/ perceived social isolation was associated with…
	Cross-sectional Studies
Layden et al., 2016	- increased brain-wide functional connectivity (FC) in right central operculum and right supramarginal gyrus<br>- increased FC in cingulo-opercular network (e.g. in bilateral insula/ frontoparietal opercula)<br>- reduced FC between cingulo-opercular network and right middle/ superior frontal gyrus
Inagaki et al., 2017	- increased ventral striatum activity when viewing a close other (vs. a stranger)
Kanai et al., 2012	- less gray matter volume (GMV) in left posterior superior temporal sulcus (pSTS)<br>- weaker activation in ventral striatum when viewing pictures of pleasant social events (vs. pictures of pleasing nonsocial events)<br>- weaker activation in bilateral temporoparietal junction (TPJ) to unpleasant social pictures of people (vs. unpleasant pictures of objects)
Kong et al., 2014	- greater regional gray matter volume (rGRV) in left dorsolateral prefrontal cortex (DLPFC)
Tian et al., 2017	- altered interactions between resting-state networks<br>- weaker causal flow from dorsal attentional network (DAN) to ventral attentional network (VAN)<br>- decreased causal flow from affective network (AfN) to VN (visual network) inducing stronger VN activity<br>➔ decreased top-down control from higher networks leads to stronger activation in lower sensory networks
Ti et al., 2018	- greater spontaneous neural activity in right inferior temporal gyrus (ITG)
Wong et al., 2016	- loneliness-related sub-network identified: structural brain networking in regions within the salience network (i.e. amygdala) + additional component that connects left globus pallidus with temporal and occipital regions<br>- increased connectivity within default mode network and cortico-striatal network<br>➔ aberrant networking in subcortical area while processing negative stimuli<br>➔ perceived loneliness plays unique role in relation to negative affective processing (in late-life depression)

D'Agostino, Kattan, Canli, 2018	- older adults were less lonely compared to younger and dwelled longer on faces, regardless of valence<br>- no association between loneliness and VS activation to social pictures of strangers in either age group<br>- no association between social network size and amygdala activation to social stimuli

Longitudinal Studies

Ehlers et al., 2017	- change in regional brain volume mediates effects of changes in social support and stress on change in perceived loneliness<br>- larger baseline amygdalae associated with greater decreases in loneliness (mediated by reductions in stress)<br>- larger baseline PFC volume was associated with greater reductions in stress (mediated by greater increase in social support)
